# 

**Published:** 1908-05

**Authors:** 


					﻿Sixty-third Year.
Vol. LXII	MAY 1908.	No. 10
BUFFALO
MEDICAL JOURNAL
Established 1845 by AUSTIN FLINT M. D.
EDITOR:
WILLIAM WARREN POTTER, M.D.
ASSISTANT EDITORS:
WILLIAM C. KRAUSS, M. D.	NEESON W. WILSON, D.
ASSOCIATE EDITORS:
JAMES WRIGHT PUTNAM, M. D. ERNEST WENDE, M. D.
JOHN PARMENTER, M. D.	JOHN A. MILLER, Ph. D.
MAUD J. FRYE, M. D.
				

